# Using mid-upper arm circumference ***z***-score measurement to support youth malnutrition screening as part of a global sports and wellness program and improve access to nutrition care

**DOI:** 10.3389/fnut.2024.1423978

**Published:** 2024-08-12

**Authors:** Amy R. Sharn, Raissa Sorgho, Suela Sulo, Emilio Molina-Molina, Clara Rojas Montenegro, Mary Jean Villa-Real Guno, Susan Abdel-Rahman

**Affiliations:** ^1^Global Medical Affairs and Research, Abbott Nutrition, Columbus, OH, United States; ^2^Center for Wellness and Nutrition, Public Health Institute, Sacramento, CA, United States; ^3^Global Medical Affairs and Research, Abbott Nutrition, Chicago, IL, United States; ^4^Research and Development, Abbott Nutrition, Granada, Spain; ^5^Universidad del Rosario-Escuela de Medicina, Bogotá, Colombia; ^6^Ateneo de Manila University School of Medicine and Public Health, Pasig, Philippines; ^7^Health Data Synthesis Institute, Kansas City, MO, United States

**Keywords:** mid-upper arm circumference, pediatric malnutrition, pediatric malnutrition screening program, program evaluation, child health, nutrition programs

## Abstract

**Objective:**

Historically, mid-upper arm circumference (MUAC) has been instrumental to identifying malnutrition in children under 5 years living in resource restricted settings. Less attention is directed to at-risk, school-aged youth. Updated and validated pediatric age- and gender-specific MUAC growth curves expand malnutrition screening opportunities (2 months-18 years) including overweight/obesity. An innovative partnership was created to integrate MUAC *z*-score measurement trainings and screenings in the Real Madrid Foundation's (RMF) Social Sports Schools (S^3^) program, which provide sports and wellness programming to under-resourced communities. This work aimed to investigate the feasibility of leveraging non-healthcare professionals (non-HCPs) to identify malnutrition risk as part of RMF S^3^.

**Methods:**

This global, two-part program on malnutrition risk identification included training adult facilitators and screening children attending RMF S^3^. RMF facilitators were trained with didactic lectures on malnutrition, and practical hands-on learning of proper MUAC *z*-score tape measurement. Aggregate data on facilitators and the number of times to correctly administer the MUAC *z*-tape were recorded. Aggregate data on child malnutrition risk screenings were collected.

**Results:**

Nine countries participated representing Europe, Pacific Asia, Africa, Latin America, and North America. In total, 143 RMF facilitators were trained, and 318 children were screened across 11 sites. More than half of facilitators were male (56%, *n* = 80), and majority were coaches (41.3%, *n* = 59), followed by staff (25.2%, *n* = 36), and volunteers (16.1%, *n* = 23). Facilitator attempts ranged from 1 to 4 times for proper MUAC *z*-score administration with mean 2.12 (± 0.86). There were no significant differences for attempts among RMF facilitator types (*p* = 0.10). Sixteen percent (*n* = 51) of children screened were recommended for HCP referral, with concentrations in Pacific Asia (68%, *n* = 35), Latin America (24%, *n* = 12), and Africa (8%, *n* = 4).

**Conclusions:**

Findings from our sample demonstrate that integration of MUAC *z*-score based malnutrition risk screening within community sports and wellness programming among non-HCPs is feasible, and that some regions with less frequent access to routine health care may experience greater benefit from these programs. Equipping non-HCP facilitators in community sports and wellness programs with training on malnutrition screening provides a means to meet under-resourced families where they live to begin conversations around malnutrition risk with the hope of establishing additional pathways to care.

## Introduction

International health leaders emphasize every child's right to good nutrition (1). Well-nourished children can grow and develop to meet their full potential. They are better able to experience healthy lives, to learn and take part in their communities, and to thrive throughout life (1). There have been marked advances in maternal and child nutrition, including a one-third lowered proportion of children suffering from stunting over the past decade (2), yet the tolls of malnutrition (as undernutrition and nutrient deficiencies) continue to threaten the ability of many children to survive and thrive. In 2022, the global number of children under 5 years old with stunted growth was 148 million (22.3% of under 5 years), and 45 million with wasting (6.8%) (3). Such malnutrition is responsible for nearly half of all deaths in children younger than 5 and causes cognitive impairment in many who survive (4, 5).

The causes of pediatric malnutrition as nutritional inadequacy are many, but the primary cause is having limited access to sufficient healthy foods in under-resourced communities, especially in developing countries (4). Underlying social determinants of health that contribute to acute pediatric malnutrition include: economic instability, social instability with limited access to quality education (especially for the child's mother), and poor access to healthcare and other supportive programs in the community (6). Secondary pediatric malnutrition usually occurs as disease-related malnutrition, especially with severe acute conditions such as cancer (7, 8), infections (9), or chronic conditions such as allergies (10), congenital heart disease, and chronic diseases of the kidneys, liver, or intestine (11, 12).

A global strategy to address the problem of pediatric malnutrition begins with broad-scale screening of children for evidence of poor nutrition. However, more than half of the world's population lacks access to essential health services (2). Historically, mid-upper arm circumference (MUAC) has been instrumental to identifying malnutrition in children under 5 years living in resource restricted settings and has been used by both non-health care professionals (HCPs) and HCPs (13–19). MUAC has been validated to be used to screen for malnutrition risk by non-HCPs in resource-restricted settings and to be used as a single indicator for pediatric malnutrition when used by HCPs by the Academy of Nutrition and Dietetics and the American Society for Parenteral and Enteral Nutrition (13). Consequently, finding alternative ways to identify and address malnutrition risk has become imperative. Measurement of a child's MUAC and comparison of the results with standard norms is a quick and validated way to detect malnutrition risk (14, 15, 17). Color-coded MUAC insertion tapes with fixed thresholds at 11.5 and 12.5 cm simplify the assignment of malnutrition risk, especially for non-HCPs. However, their application is limited to children from 6 months to 5 years of age, and they identify only those children with the most severe malnutrition, overlooking a significant proportion of children who would benefit from nutritional repletion.

Many have called for a refinement of MUAC thresholds to incorporate age- and gender-specific normative data (i.e., *z*-scores). Nutrition-centric organizations also began recommending the inclusion of MUAC *z*-scores in the evaluation of malnutrition risk for all children. To address this need, investigators at The Children's Mercy Hospital (Kansas City, Missouri, USA) generated MUAC growth curves and developed the MUAC *z*-score tape to expand the population of children that can be screened for malnutrition (2 months to 18 years) without the use of a pediatric growth chart, while more accurately triaging the underlying severity of their malnutrition.

To achieve a better future for all, the United Nations established the Sustainable Development Goals (SDGs) as a blueprint. The SDGs target global challenges of poverty, inequality, wars and injustices, climate change, and environmental degradation. As related to childhood nutrition and wellbeing, 3 of these goals are relevant: Goal 2 is Zero Hunger, Goal 3 is Good Health and Wellbeing, and Goal 17 is to Strengthen Implementation and Revitalize Global Partnerships that can support sustainable development (20). This work presents early findings from a pilot feasibility program designed to impact pediatric malnutrition with strategies that pursue SDGs 2, 3, and 17. While pediatric malnutrition represents a double burden as under- and over-nutrition (21), the focus of the pilot feasibility program described in this paper is undernutrition.

Pediatric anthropometric assessments have mostly been performed by professionals in healthcare settings; however, recent programs have shown that lay people including community volunteers and family members can be readily trained to perform MUAC screening in their communities (22). Utilizing MUAC *z*-score tapes also provides an opportunity for measurement where barriers such as access to equipment, electricity, or uneven surfaces restrict the use of scales and stadiometers for weight and height/length measures. By equipping non-healthcare professionals (non-HCPs) to screen for malnutrition, there is an immense opportunity to build self-efficacy of community leaders to begin conversations about the importance of good nutrition and its impact on child health and growth.

We presently report on a two-part program for use of the new MUAC *z*-score tape as an innovation to identify children with malnutrition or who are at risk—a first step toward accelerating pathways to care for children who need nutritional support. Here we describe an exemplary partnership between a public health group (Public Health Institute Center for Wellness and Nutrition, Sacramento, CA, USA), a healthcare industry company via its Center for Malnutrition Solutions (Abbott Laboratories, Chicago, IL, USA), and a service foundation for youth sports (Real Madrid Foundation, Madrid, Spain). The partners implemented a model program to: (1) train Real Madrid Foundation football coaches, volunteers, and staff on the importance of good nutrition and how to properly use the MUAC *z*-score tape; and (2) conduct MUAC *z*-score-based screening for risk of undernutrition or nutrient deficiencies.

Specifically, program leaders trained non-HCP community sports facilitators (staff, coaches, volunteers) to screen children with MUAC *z*-score tapes in RMF S^3^ programs for malnutrition risk. Malnutrition risk was based on a MUAC *z*-score 2 or more standard deviations below (undernutrition) or over (overnutrition) the mean for age. *Z*-scores greater than the mean (overweight, obesity) were also accessible to participants but this program focused on undernutrition.

### Specific objectives of the malnutrition training and screening program

The program investigated the feasibility of a broad-scale program to: (1) train community coaches, staff, and volunteers on the importance of nutrition and how to screen for malnutrition risk with MUAC *z*-score tapes; and (2) screen for malnutrition risk using MUAC *z*-score tapes in children living in under-resourced communities worldwide as part of community sports and wellness programming. The long-term goal is to improve health outcomes in children by equipping points of contact in their everyday life with the skills to offer identification, education, and, where necessary, medical referral for undernutrition.

## Materials and methods

### Program framework

As a framework for endorsing and enforcing pediatric nutrition care, the current pilot feasibility program represents a working model with a 3-party partnership.

First, the *Public Health Institute's Center for Wellness and Nutrition* (*PHI CWN*) is a program for improving health, equity, and wellness by putting new research into action with campaigns and partnerships in communities worldwide, especially for populations that are vulnerable (23). *PHI CWN* uses education, engagement, and public health policy changes to facilitate health access for all.

Second, the healthcare industry partner is the *Abbott Center for Malnutrition Solutions* (ACMS) (24), which seeks to develop a network of community-based partners and to build a system that can help improve health and nutrition in communities around the world.

Third, The *Real Madrid Foundation* (RMF) (25) is the outreach arm of the *Real Madrid Football Club* that implements humanitarian and social projects worldwide. Through its Social Sports Schools (S^3^), RMF provides children living in under-resourced communities with afternoon and weekend programming in a supportive environment; RMF S^3^ are active in more than 80 countries.

### Part 1: RMF facilitator training

Part one of the Nutrition Screening Program's goal was to increase the knowledge and awareness of the RMF facilitators (S^3^ staff, coaches, and volunteers) on the topics of healthy child nutrition status to improve the nutrition of the S^3^ enrollees and assess training feasibility with aggregate data. All participating RMF facilitators were recruited, were adults over the age of 18, who consented to participate in the training, as part of their signed agreement with the RMF for involvement in the S^3^. The pilot feasibility program only worked with staff currently recognized and contracted/in agreement with the existing program. Training by ACMS and Abbott Nutrition Medical Affairs and/or PHI CWN of RMF facilitators to effectively conduct nutrition screening using the MUAC *z*-score (measuring) tape allowed the RMF facilitators to identify children at risk for malnutrition, open dialogue with families, and encourage them to seek professional care around the issue, if necessary. Through the implementation of nutrition screening, ACMS, PHI CWN, and RMF created an evidence-based model that can be shared with other organizations and future partners to increase awareness around malnutrition risk while demonstrating a pathway to care.

The implementation of the pilot feasibility program included preparing and developing training materials on the importance of nutrition and malnutrition screening along with designing and delivering trainings to the RMF facilitators in English and local languages as needed. RMF facilitators were trained by the ACMS and PHI CWN to use the MUAC *z*-score tape to measure and interpret the MUAC *z*-score. RMF facilitators were trained to collect MUAC *z*-scores by marking the midpoint of the child's upper arm, located halfway between the acromion process of the shoulder and olecranon. The MUAC *z*-score measuring tape was next wrapped around the child's arm at this midpoint, the MUAC was measured to the nearest 0.1 cm, and the MUAC *z*-score range determined by direct visual inspection of the tape. Facilitators were also trained on aggregate data collection, data storage, child assent, and communicating program details with the children's caregivers.

This training created a nutrition screening program to be implemented and scaled up by RMF across its S^3^ internationally. The materials and trainings were offered in English and in the relevant local/national language by a verified translator vendor based at the location of the S^3^. Following training, the RMF facilitators received certificates of participation, outlining the training they completed, and the skills gained. Upon completion of the training, RMF facilitators were able to utilize their skills to discuss nutrition screening with S^3^ enrollees, use the MUAC *z*-score tape, and routinely screen the nutritional status of their pediatric S^3^ enrollees, as well as document the aggregate data.

### Part 2: pediatric MUAC *z*-score screening and nutritional risk determination

**Figure 1:**
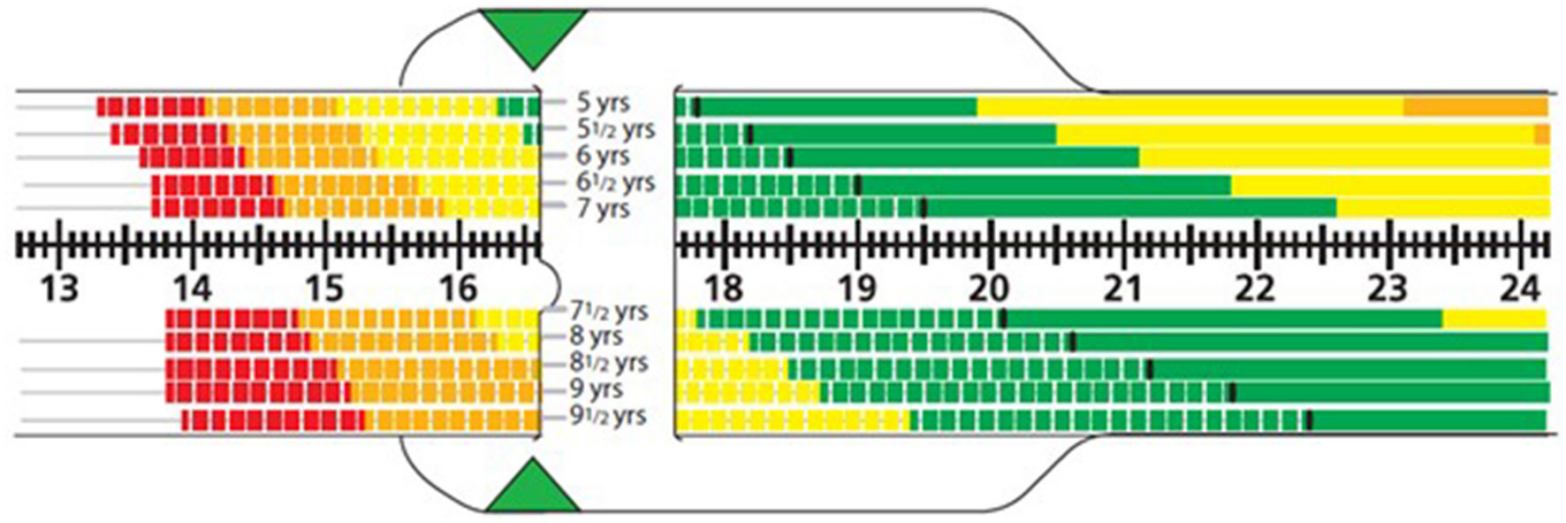
Sample reading from one side of the MUAC *z*-score tape. The example shows a MUAC reading 16.6 cm. For a 7-year-old child, this corresponds to a hashed yellow region, i.e., mild malnutrition; whereas a 9-year-old child would be in the range for moderate malnutrition (hashed orange region).

MUAC *z*-score (color and shading) was measured by RMF facilitators using the flexible Abbott MUAC *z*-score tape for the appropriate age range as part of a pilot feasibility program (Abbott Laboratories, Chicago, USA) (24). Based on the measured circumference and age of the child, a *z*-score range was read and recorded from the tape based on color and shading (Figure 1). Malnutrition risk was signaled by the MUAC *z*-score and by color coding and shading (hashed vs. non-hashed). Scores from +1 to 0 to −1 are in the normal range (green). Mild, moderate, and severe levels of undernutrition, respectively, were defined as MUAC *z*-scores of −1 to −2 (hashed yellow); −2 to −3 (hashed orange); and −3 to −4 (hashed red) as laid out by the Academy of Nutrition and Dietetics and the American Society for Parenteral and Enteral Nutrition (13).

### *Pathway to care* for children with MUAC *z*-scores indicative of malnutrition risk

Following screening, children who fell in the categories of moderate (orange) to severe (red) malnutrition risk, were referred for follow-up with an HCP for further assessment and treatment.

### Site selection and participant recruitment

Sites were selected in partnership with RMF based on site enrollment and staffing capabilities to represent the global reach of RMF's S^3^ programming in under-resourced communities. Adult participants were recruited if appointed to working or volunteering for RMF's S^3^ programming. Child participants aged 5–17 years were recruited as part of their enrollment in S^3^.

### Analyses

Descriptive statistics for continuous data (mean with SD or median with range) and categorical data (*N*%) were calculated for respondents' characteristics (facilitator type, number of attempts, number of children screened, number of children with malnutrition risk). To reflect differences between groups Student *t*-tests were performed and *p*-values <0.05 were considered statistically significant. All analyses were performed via Microsoft Excel.

### Ethical review

This pilot feasibility program protocol was reviewed and approved as exempt by the Institutional Review Board of Public Health Institute in Sacramento, CA, USA.

## Results

**Figure 2:**
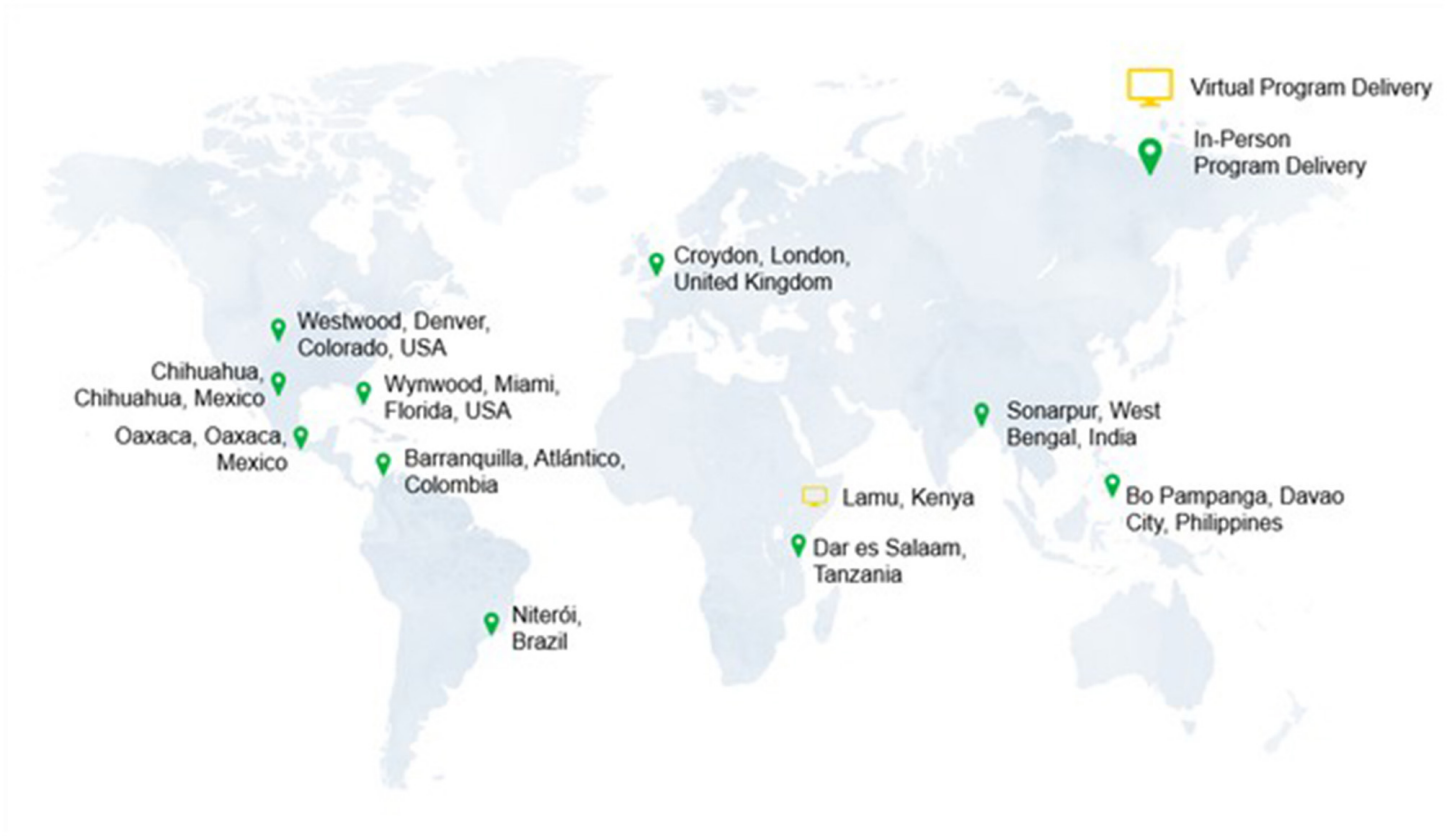
ACMS-RMF training and screening for pediatric malnutrition was conducted in nine countries (11 sites).

This global pilot feasibility training and screening program took place at 11 sites in nine countries: Brazil, Colombia, India, Kenya, Mexico, Philippines, Tanzania, United Kingdom, and United States (Figure 2).

**Figure 3:**
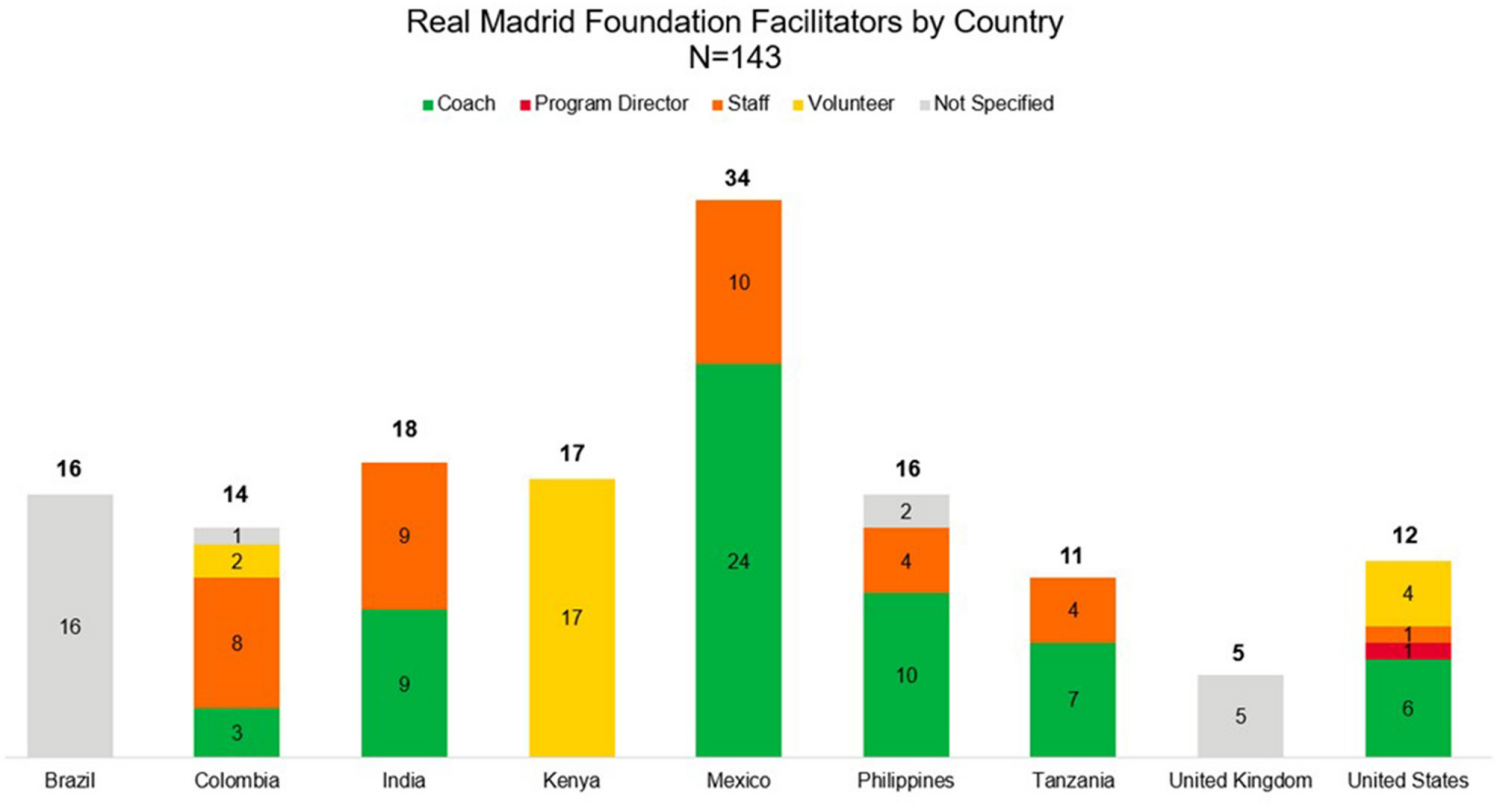
RMF facilitators by country. This figure shows the number of facilitators trained by country and their type; the total number trained was *N* = 143.

In this pilot feasibility program, a total of 143 RMF facilitators were trained for a total of 286 training hours, with the largest number of trained facilitators (*n* = 34) Mexico (Figure 3). All training programs were conducted in person except for 1 in Lamu, Kenya. Of these facilitators, 41.3% were coaches, 25.2% were staff, 16.1% were volunteers, and 16.8% were not specified. One program administrator was also trained, representing 0.7%.

After training, facilitator attempts to determine a MUAC *z*-score correctly ranged from 1 to 4 attempts, with a mean of 2.12 (± 0.86 standard deviation). There were no significant differences in attempts by RMF facilitator types (coaches, staff, volunteers).

Of the children screened for malnutrition by MUAC *z*-score measures and *z*-score determinations, a total of 16% were identified to be at malnutrition risk and were referred to a healthcare provider by RMF facilitators for further evaluation and potential treatment if indicated (Table 1). The proportion of screened children with undernutrition varied widely from 0% in the UK and the USA to 50% in India; child screening data from Kenya and the Philippines were not collected as part of training day activities. The highest prevalence of malnutrition risk was children of test communities in India (50%), Tanzania (16%), and Colombia (12%).

**Table 1:** Child nutrition screening results by country.

**Country**	**Total screened, *n***	**Total at risk/referred, *n* (%)**
Brazil	50	4 (8)
Colombia	25	3 (12)
India	70	35 (50)
Kenya	0	0 (0)
Mexico	105	5 (5)
Philippines	0	0 (0)
Tanzania	25	4 (16)
United Kingdom	19	0 (0)
United States	24	0 (0)
TOTAL	318	51 (16)

## Discussion

### Summary of key results

Findings from our unique 3-partner program demonstrated that malnutrition risk in children living in under-resourced communities worldwide could be identified by use of a simple and straightforward MUAC *z*-score tape procedure as part of community sports and wellness programming. Importantly, non-HCP RMF S^3^ facilitators, could be readily trained to conduct the MUAC *z*-tape screenings. Following training, the mean number of attempts to reach a trainer approved MUAC determination was just over two tries.

Results from the nine participating countries showed that the highest incidence of malnutrition risk were children of RMF sample communities in India, Tanzania, and Colombia. These findings are similar to reported malnutrition rates of young children under 5 within these countries. In India, 34.7% of children have stunting, 17.3% have wasting, and 1.6% have overweight (26). Estimates within Tanzania include 31.8% of children with stunting, 3.5% with wasting, and 2.8% with overweight (27). Throughout Colombia, estimates indicate 12.7% of children have stunting, 1.6% have wasting, and 5.7% have overweight (28).

However, data are limited on malnutrition rates among school-aged children who were the focus of this pilot feasibility program (ages 5–17 years old) due to global public health attention to young children who are more vulnerable to malnutrition-related complications (2, 3). However, research studies among school-aged children occurring in India (29, 30), Tanzania (31, 32), and Colombia (33–35), report similar results with high incidences (>10%) of pediatric malnutrition. And in Colombia, a study found that among girls, overweight/obesity, stunting, and anemia were experienced more frequently among those with lower socioeconomic status (35).

Although this program did not track socio-demographic or economic data, India, Tanzania, and Colombia are experiencing challenging economies with gross domestic product (GDP) per capita being $2,410.90, $1,192.80, and $6,624.20, respectively (36). At the population level, India (12.92%) (26), Tanzania (44.95%) (27), and Colombia (6.6%) (28) have a significant proportion that live with <$2.15 per day. Challenging economic environments can also contribute to other worsening components of social determinants of health, which may impact the quality, access, and availability to nutrient dense foods to support child growth. This may have contributed to the higher incidences of malnutrition at RMF S^3^ locations in our sample.

While the number of children screened in this pilot feasibility program were limited, the results indicated good potential for such screening where routine health care evaluations may be lacking and there are continued challenges among social determinants of health. We found that nutritional screening of children participating in community sports and wellness programs could be readily accomplished by their non-HCP coaches and by affiliated staff members and volunteers. Successful training for MUAC *z*-score tape use was readily accomplished by training of leaders in the RMF S^3^ community sports and wellness programming.

### Prior efforts using MUAC for nutrition screening in pediatric populations

The early identification of malnutrition has been the focus of numerous studies to identify and treat nutritional deficits. MUAC measures are considered a reliable proxy for muscle mass, and MUAC is recommended to identify malnutrition in children, especially with updated parameters that account for child growth (16). However, fixed MUAC cut-off points are inadequate and needed to be adjusted. A study conducted in India (2,650 children, ages 6–59 months) identified that a cut-off point <12.8 cm (well in excess of the reported 11.5 cm) was needed to ensure that all children at risk for malnutrition were identified (sensitivity 74.1%; specificity 93.2%; Youden index 0.67) (16).

In another study of Ethiopian children under 5 years of age (*n* = 25,755), researchers also found that the standard World Health Organization's MUAC cut-off point of 11.5 cm did not identify all children at risk for malnutrition and recommended that MUAC <12.5 cm should be used for community screening (17). These researchers further recommended development of gender- and age-sensitive MUAC scores (17). Another study of Sri Lankan school-aged children (ages 5–10 years, *n* = 538) utilized MUAC to identify malnutrition and to determine cutoff values for thinness, stunting, overweight, and obesity (15). This pilot feasibility program concluded that MUAC was easy-to-administer and should be used for the ongoing assessment of nutritional risk status and growth in school-aged children (15).

It is critical to enhance adoption and routine use of simple screening measures such as MUAC *z*-score worldwide. Bliss et al. conducted a systematic review (22 studies) on the use of MUAC by caregivers and community health workers to identify malnutrition (children ages 6–59 months) (37). Results of the review revealed that caregivers could accurately detect severe acute malnutrition (SAM) in their own children (37). Results further showed that when community health workers identified SAM using MUAC, they also provided nutritional treatments (37). In a UNICEF-conducted review of the effectiveness of the Family-MUAC program in West and Central Africa (using published and unpublished data), results showed that mothers and caregivers were able to perform MUAC measurements effectively (22).

Taken together, these studies highlight that the MUAC tape can be used to identify the double burden of malnutrition (under- and over-nutrition) in community settings across the globe where malnutrition may present differently. Thus, community-based strategies for MUAC-identified malnutrition along with provision of nutritional treatment is expected to improve both health and healthcare cost outcomes for children worldwide; future studies need to quantify benefits such as nutrition-focused quality improvement programs that measure the impact of nutrition screening and intervention with appropriate nutrition supplements on health, clinical, and economic outcomes (22).

### Strengths and limitations of the current pilot feasibility programs its findings

Our program results indicated that awareness of pediatric nutrition could be raised when children with malnutrition risk were identified from community sports and wellness programs. The study specifically demonstrated the feasibility of implementing a nutritional screening program in communities where food and healthcare resources may be limited, thus bringing care to families and children who need it most. The program also showed that non-HCP facilitators allied with the sports programs (coaches, staff, affiliated volunteers) could be successfully trained to use a MUAC *z*-score tape as a tool to identify children who may be at risk for malnutrition. Once children with malnutrition risk were identified, the facilitators had the skills to begin conversations with caregivers about the importance of nutrition in childhood and encourage follow-up with a healthcare professional for further evaluation and treatment if indicated. Community leaders and caregivers are thus alerted to the importance of taking action to improve access to food and healthcare resources.

However, our pilot feasibility program also had limitations. We used convenience sampling of sports-affiliated facilitators, which are not representative of a broader population of facilitators working with children worldwide. Our pilot feasibility program had a limited number of participants (facilitators trained and children screened) and included school age children that malnutrition rates are not as frequently tracked as their under 5 years old peers. This limits our ability to compare our results with current malnutrition prevalence data.

Our pilot feasibility program also did not track socio-demographic information of facilitators and participating children as this was a program evaluation and therefore individual-level data were not collected. Therefore, such information could not be used in the analysis to better understand differences in RMF facilitator groups or risk profiles of children with different socio-demographic characteristics.

This pilot feasibility program was limited in using MUAC measures without collecting additional anthropometrics to assess proper pediatric growth. Uneven ground and limited resources and access to locations can inhibit the proper measurement and transportation of equipment to measure anthropometrics in the community. Additionally in the pediatric population, using anthropometrics outside of MUAC as a proxy for malnutrition status also requires the use of pediatric growth curves that require clinician training—something worth exploring in a future study. No MUAC or nutritional changes after the first identification are reported and pathways of care were limited to a referral to an HCP. Future studies should employ study designs to allow for collection of child level socio-demographic, MUAC, nutritional and clinical data longitudinally to assess the effectiveness of similar programs in widening access and improving nutrition care. This is in scope for future analysis of the effectiveness of RMF Malnutrition Screening Program.

### Next steps

Childhood malnutrition still affects many millions of children worldwide. To build the impact of the RMF Malnutrition Screening Program and others like it, we call for expert guidance on pathways for nutrition care within the community that meet individuals where they live and play to assist in alleviating some of the burdens of inadequate social determinants of health. Future work should build collaborations with community programs that are the experts of their lived experience to inform meaningful screening, education, intervention, and follow-up nutrition programs. Programs that focus on malnutrition should focus on incorporating a multi-pronged approach to impact multiple socioecological levels concurrently, with stratifications for treatment dependent on malnutrition severity. We further emphasize the importance of a systematic approach to nutrition care—screening for malnutrition risk in school-aged children, diagnosing malnutrition by an HCP, treating/feeding to mitigate consequences, and preventing future occurrence or recurrence (13, 38). This overall approach serves as a reminder that children, and people of all ages, need consistent and good nutrition to remain healthy across the lifespan.

To enhance nutritional care for children, we call for the following actions:

- Scale up: expand the number of trainers, facilitators, and screenings conducted worldwide to widen access to care.
- Scale up longitudinal assessment: design and conduct large and prospective studies to follow children over time after identification of malnutrition and provision of nutritional support and inform collection of nutrition, clinical and economic outcomes to demonstrate how nutrition care can generate clinical, economic, humanistic, and societal value for the children, their families, their communities, and societies at large.
- Scale out: Screen new populations of children through community programs outside of sports and wellness programs (39).

We have described a unique partnership between a public health group (Public Health Institute *Center for Wellness and Nutrition*), a private sector organization (*Abbott Center for Malnutrition Solutions)*, and a foundation for youth sports and wellness initiatives (*Real Madrid Foundation*). These partners implemented a model program to train sports team coaches and community volunteers to conduct MUAC-based screening for risk of malnutrition or nutrient deficiencies. Pilot feasibility program results showed that such a training/screening program was feasible. The present report on our pilot feasibility program shows that non-HCP leaders of community youth sports and wellness programs can be readily trained to use the MUAC *z*-score tape method to screen for malnutrition in program participants.

The clinical implications of our findings represent a call-to-action on a clear need for uptake of methods that promote child nutrition and health worldwide (40). The program described is a unique partnership between a public health group and its affiliated industry and private partners. Such a program is expected to contribute to achieving the United Nation's world goals of Zero Hunger, Good Health and Wellbeing, and Stronger Global Partnerships to support sustainable development (1, 3).

## Data availability statement

The data supporting the conclusions of this study are available upon reasonable request.

## Author contributions

ARS: Writing – original draft, Writing – review & editing. RS: Writing – review & editing. SS: Writing – original draft, Writing – review & editing. EM-M: Writing – review & editing. CR: Writing – review & editing. MV-RG: Writing – review & editing. SA-R: Writing – review & editing.
